# Author Correction: Vitamin B12 induces memory of predation through vitellogenin provisioning

**DOI:** 10.1038/s41467-026-77839-9

**Published:** 2026-09-21

**Authors:** Shiela Pearl Quiobe, Ata Kalirad, Raphaela Zurheide, Hanh Witte, Christian Rödelsperger, Ralf J. Sommer

**Affiliations:** Department for Integrative Evolutionary Biology, Max Planck Institute for Biology Tübingen, Tübingen, Germany

**Keywords:** Epigenetics, Epigenetic memory

Correction to: *Nature Communications* https://doi.org/10.1038/s41467-026-71494-w, published online 09 April 2026

In the version of this Article initially published, Fig. 1b contained two errors. The genus name “Pristionchus” was misspelled, and the y-axis label was incorrectly given as “% Predatory worms” instead of “Predatory worm counts”. The figure is now updated in the HTML and PDF versions of the article.

