# Supplementary material for: Vitamin B12 induces memory of predation through vitellogenin provisioning
Source: Nat Commun. 2026 Apr 9;17:3408. doi: 10.1038/s41467-026-71494-w (PMC13068944; doi:10.1038/s41467-026-71494-w)
Supplement: Supplementary file 1 — Supplementary information [file 41467_2026_71494_MOESM1_ESM.pdf]

Supplementary information:

Vitamin B12 induces memory of predation through vitellogenin provisioning

Shiela Pearl Quiobe[1], Ata Kalirad[1], Raphaela Zurheide[1], Hanh Witte[1], Christian Rödelsperger[1] & Ralf J. Sommer[1*]

[1]Max Planck Institute for Biology Tübingen, Department for Integrative Evolutionary Biology, Max-Planck Ring 9, 72076 Tübingen, Germany

- Author for correspondence at: ralf.sommer@tuebingen.mpg.de

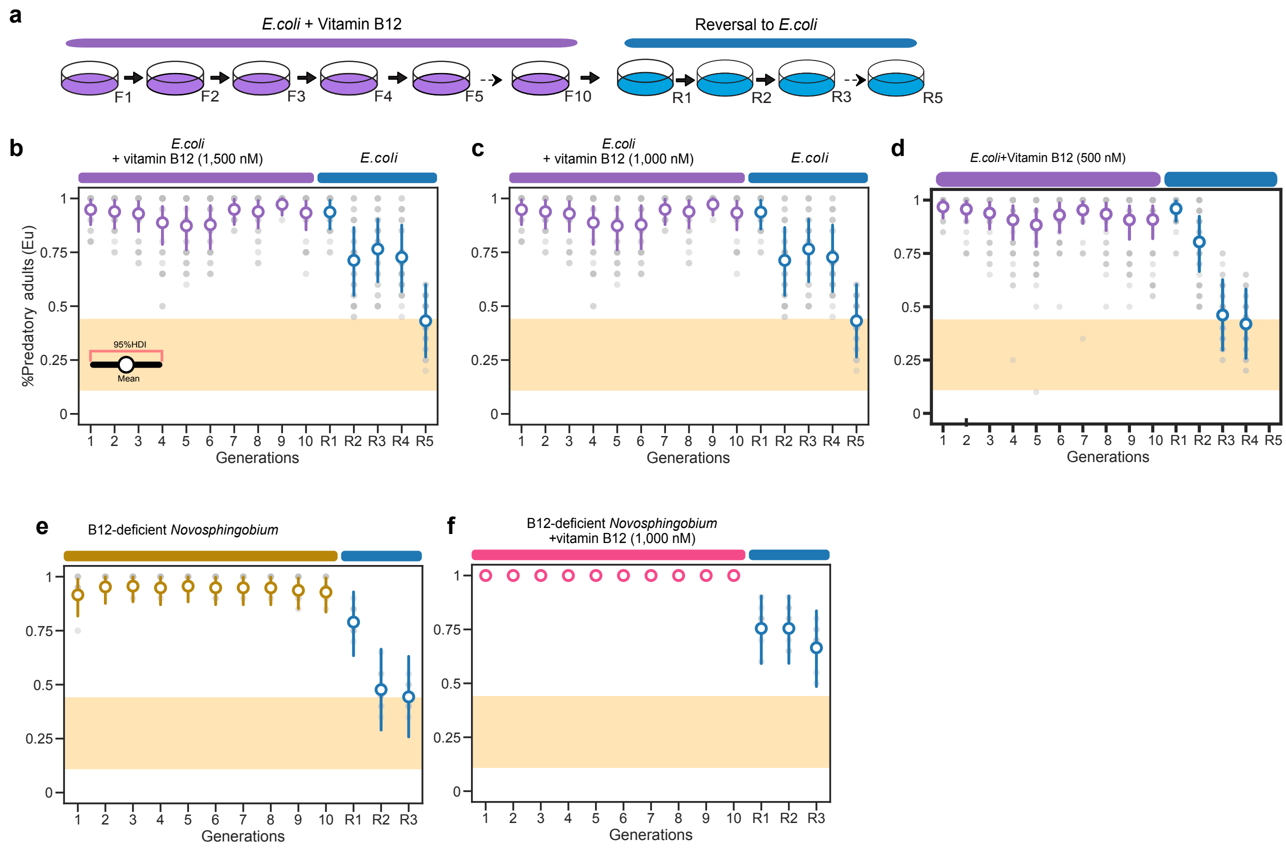

Supplementary Figure 1. Induction of the predatory mouth form by vitamin B12- supplemented *E. coli*, *Novosphingobium* vitamin B12-deficient mutant and vitamin B12 rescue.

a, Schematic diagram of worms grown on *E. coli* supplemented with vitamin B12 for 10 generations and reversal to un-supplemented *E. coli*. **b-d,** Mean probability of the predatory mouth form after 10 generations of vitamin B12 supplementation with 1,500 nM (b), 1000 nM (c) and 500 nM (d) vitamin B12 and reversal to un-supplemented *E. coli*. **e,** Mean probability of the predatory mouth form after 10 generations on a *Novosphingobium* vitamin B12-deficient mutant and reversal to *E. coli*. **f,** Mean probability of the predatory mouth form after 10 generations on a *Novosphingobium* vitamin B12-deficient mutant supplemented with exogenous vitamin B12 and reversal to *E. coli*. Final mouth-form frequencies are the mean of at least 10 biological replicates (n = 20 animals per plate). Points show the mean probability of developing the Eu morph, with error bars representing the 95% HDI from the Bayesian model. The yellow area indicates the established RSC011 baseline response on *E. coli*, averaged over 101 generations.

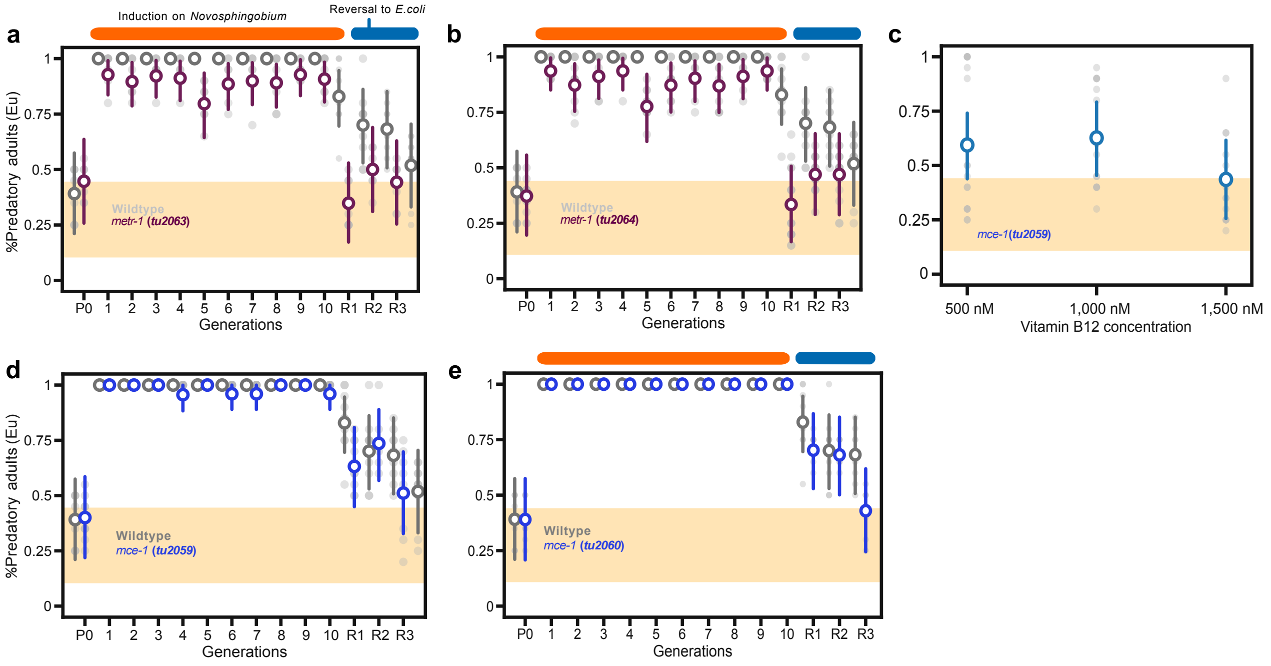

Supplementary Figure 2. *Ppa-metr-1* and *Ppa-mce-1* response on *Novosphingobium* and reversal to *E. coli*.

a,b, Mean probability of the predatory mouth form in *Ppa-metr-1* mutant animals on *Novosphingobium* after 10 generations of exposure and reversal to *E. coli*. **c,** Mean probability of the predatory mouth form in *Ppa-mce-1* mutant animals on vitamin B12-supplemented *E. coli* plates. **d,e,** Mean probability of the predatory mouth form in *Ppa-mce-1* mutant animals on *Novosphingobium* after 10 generations of exposure and reversal to *E. coli*. Final mouth-form frequencies are the mean of at least 5-10 biological replicates (n = 20 animals per plate). Points show the mean probability of developing the Eu morph, with error bars representing the 95% HDI from the Bayesian model. The yellow area indicates the established RSC011 baseline response on *E. coli*, averaged over 101 generations. More details about the molecular lesions are listed in Supplementary Table 1.

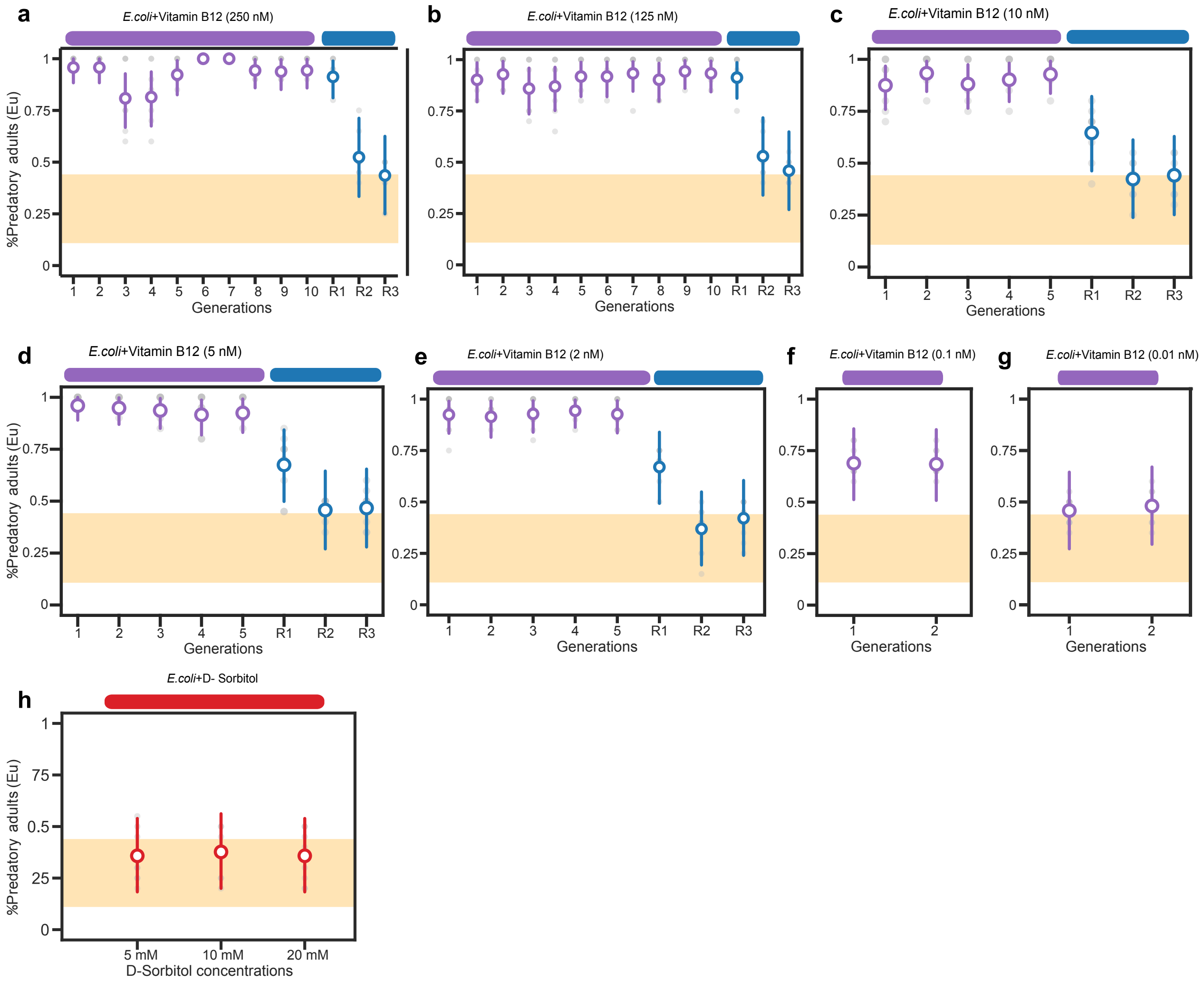

Supplementary Figure 3. Vitamin B12 supplementation on the induced predatory mouth form and its maternal effect after reversal on *E. coli*.

a-b, Mean probability of predatory mouth-form on **(a)** 250, **(b)** 125 nanomolar (nM) of vitamin B12 supplementation for 10 generations and subsequent exposure to un-supplemented *E. coli*. **c-e**, Mean probability of predatory mouth-form on **(c)** 10, **(d)** 5, **(e)** 2 nM of vitamin B12 supplementation for 5 generations and subsequent exposure to un-supplemented *E. coli.* **f-g,** Mean probability of predatory mouth-form on **(f)** 0.1, **(g)** 0.01 nM. of vitamin B12 supplementation for 2 generations. **h,** Mean probability of predatory mouth-form on sorbitol-supplemented *E. coli* plates. Final mouth-form frequencies are the mean of at least 5-10 biological replicates (n = 20 animals per plate). Points show the mean probability of developing the Eu morph, with error bars representing the 95% HDI from the Bayesian model. The yellow area indicates the established RSC011 baseline response on *E. coli*, averaged over 101 generations.

Alleles	Gene ID; *C. elegans* best hit	Molecular lesions	sgRNA(PAM)	Forward primer	Reverse primer
tu2063	RSC011000010081; *metr-1*	10 bp net insertion	AGAAATGAGACCATTCGTAG(AGG)	GAGAACCACACTCCGATACG	CAACTTGACTACGAGCTATTG
tu2064		55 bp net insertion			
tu2059	RSC011000034399; *mce-1*	13 bp insertion	CGCCACTCCGGACATCGAGA(AGG)	CGCCAATCTTACCAGAGTGA	CATTGATGTCCTTGACCTAC
tu2060		28 bp deletion			

Supplementary Table 1. Molecular lesions of *Ppa-metr-1* and *Ppa-mce-1*.

Allele	Gene ID	*C. elegans* 1:1 ortholog	Position	Reference	Variant	Mutation type	Nucleotide substitution
	RSC011000018058	*rme-2*	12582507	G	A	nonsynonym [R>K]	AGG>AAG
	RSC011000018058	*rme-2*	12582467	C	T	nonsynonym [L>F]	CTT>TTT
tu797	RSC011000018058	*rme-2*	12579999	C	T	nonsynonym [L>F]	CTC>TTC

Supplementary Table 2. *Ppa-rme-2* alleles isolated from the genetic screen by EMS mutagenesis.
