# Supplementary material for: Vitamin B12 induces memory of predation through vitellogenin provisioning
Source: Nat Commun. 2026 Apr 9;17:3408. doi: 10.1038/s41467-026-71494-w (PMC13068944; doi:10.1038/s41467-026-71494-w)
Supplement: Supplementary file 2 — Description of Additional Supplementary Files [file 41467_2026_71494_MOESM2_ESM.pdf]

Description of Additional Supplementary Files

File Name: Supplementary Data 1

Description: List of differentially expressed genes and enrichment analyses from single worm transcriptomics during *Novosphingobium* induction, vitamin B12 supplementation and methionine supplemention.

File Name: Supplementary Data 2

Description: List of differentially expressed genes from mixed stage samples during *Novosphingobium* induction and reversal to *E. coli*.
